# Induction of AhR-Mediated Gene Transcription by Coffee

**DOI:** 10.1371/journal.pone.0102152

**Published:** 2014-07-09

**Authors:** Toshio Ishikawa, Satoshi Takahashi, Koji Morita, Hiroko Okinaga, Tamio Teramoto

**Affiliations:** 1 Department of Internal Medicine, Teikyo University School of Medicine, Tokyo, Japan; 2 Teikyo Academic Research Center, Teikyo University, Tokyo, Japan; NIEHS/NIH, United States of America

## Abstract

**Background:**

Aryl hydrocarbon receptor (AhR) is classically known to be activated by xenobiotics such as dioxins and polycyclic aromatic hydrocarbons (PAHs). Although it has been reported that PAHs are contained in roasted coffee beans, in general coffee beverages are not considered to be AhR activators. We tested whether exposure to coffee would activate AhR in cultured cells.

**Methods:**

HepG2 cells stably expressing an AhR-responsive reporter gene were treated with coffee samples. Also, expression of CYP1A1, an endogenous AhR-responsive gene, was quantitated by RT-PCR and Western blotting in HepG2, Caco-2, and MCF-7 cells, after treatment with coffee. In order to obtain sensitive and reproducible results, all the experiments were performed with the cells placed in either phosphate-buffered saline (PBS) or pure serum, instead of routinely-used culture medium, whose intrinsic AhR-stimulating activity turned out to be so strong as to interfere with the analyses.

**Results:**

All the coffee samples tested robustly stimulated AhR-mediated transcription in the reporter gene assays. Of note, to what extent coffee and other AhR agonists activated AhR was different, depending on whether the experiments were done in PBS or serum. CYP1A1 mRNA was induced by coffee, in HepG2, Caco-2, and MCF-7 cells placed in either PBS or serum. CYP1A1 protein expression, which was not detected in these cells incubated in PBS, was also increased by coffee in cells placed in serum.

**Conclusions:**

By using culture medium-free experimental settings, we have shown that coffee is a strong AhR activator. Our observation may help elucidate as-yet-unrecognized effects of coffee on human health.

## Introduction

Aryl hydrocarbon receptor (AhR) is a transcription factor classically known to be activated by toxic xenobiotics such as dioxins [e.g. 2,3,7,8-tetrachlorodibenzo-*p*-dioxin (TCDD)], polycyclic aromatic hydrocarbons (PAHs) [e.g. benzo[a]pyrene (B[a]P)], etc. It can also be activated by endogenous substances [e.g. bilirubin [1]], dietary constituents [e.g. indole-3-carbinol [2]] and drug metabolites [e.g. 3-methyl-2-thiohydantoin (MTH) [3]]. The activated AhR heterodimerizes with another factor Arnt and binds to xenobiotic response elements (XREs), thereby enhancing transcription of the target genes, such as CYP1A1, CYP1A2, CYP1B1, ALDH3A1, NQO1 and UGT1A1 [4]. Many of these genes play a pivotal role in metabolizing or detoxifying harmful xenobiotics.

PAHs are usually formed by combustion of carbon-containing materials at relatively low temperatures, and it has been reported that roasted coffee beans, which are processed at a temperature of about 240°C, contain PAHs [5]. However, it has not yet been established whether coffee beverages actually stimulate AhR-mediated gene transcription. One study reported that treatment of cultured cells with coffee induced UDP-glucronosyl transferases (UGTs) through activation of AhR [6], whereas another failed to demonstrate it [7]. After all, coffee is still not generally regarded as an AhR activator. We hypothesized that the inconsistency of these two reports on coffee-induced UGT expression was ascribed, at least partly, to the interference from the AhR agonists formed in the culture medium especially after UV irradiation [8], [9]. Thus, instead of the routinely-used culture medium such as Dulbecco’s modified Eagle’s medium (DMEM) containing fetal bovine serum (FBS), we chose to use either Ca^2+^- and Mg^2+^-free phosphate-buffered saline (PBS), which should be devoid of any AhR agonist activity, or adult human serum (AHS), which in our experience had been superior to FBS-supplemented DMEM in sensitively detecting cellular AhR activation [3].

## Materials and Methods

### Chemicals and beverages

The following chemicals were purchased: MTH (Research Organics, Cleveland, OH), B[a]P (Sigma-Aldrich, St. Louis, MO), TCDD (AccuStandard, New Haven, CT), and 2-methyl-2H-pyrazole-3-carboxylic acid (2-methyl-4-*o*-tolylazo-phenyl)-amide (also known as CH-223191, which was abbreviated as “CH” in this study; Calbiochem, San Diego, CA). These chemicals were dissolved in dimethyl sulfoxide (DMSO). The following final concentrations were used in the experiments: MTH (80 µM), B[a]P (10 µM), TCDD (10^−9^–10^−6^ M), and CH (10 µM). All beverage samples (Table 1) were sugar- and milk-free, and purchased at supermarkets or grocery stores. In the experiments, they were added to the cells at the final concentration of 10% (v/v). We used 0.1% (v/v) DMSO and 10% (v/v) distilled water as a vehicle for chemicals and a control for beverages, respectively, because we had confirmed that they did not have any detectable effects in our preliminary experiments.

**Table 1:** Beverage samples tested in Figure 3.

Sample	Name	JAN
Coffee (1)	Starbucks Black Coffee	4901777237094
Coffee (2)	Tully's Coffee Barista's Black	4901085065693
Coffee (3)	UCC Black	4901201208096
Coffee (4)	Black Boss	4901777204980
Coffee (5)	Wonda Black Premium	4514603250213
Coffee (6)*	Nescafé Gold Blend	4902201330091
Coffee (7)*	Nescafé Gold Blend (decaffeinated)	4902201351348
Cocoa (1)†	Morinaga Cocoa	4902888516566
Cocoa (2)†	Van Houten Cocoa	49565102
English tea (1)	Afternoon Tea	4909411048778
English tea (2)	Sinvino Java Tea	4959127102202
English tea (3)‡	Lipton Yellow Label Tea	4902203519791
English tea (4)‡	Twinings Ceylon	4901305124827
Japanese tea (1)	*Namacha*	4909411045128
Japanese tea (2)	Roasted Tea	4901085003572
Japanese tea (3)	*Iyemon* Roasted Tea	4901777234581
Japanese tea (4)	Felice Green Tea	4936790440879
Oolong tea (1)	Oolong Tea	49152401
Oolong tea (2)	Black Oolong Tea	4901777158306
Miscellaneous (1)	*Jurokucha* (blend of 16 ingredients)	4514603240511
Miscellaneous (2)	Chestnut & Roasted Tea	4940031033522
Miscellaneous (3)	Sesame & Barley Tea	4901777235533
Miscellaneous (4)	*Kimamecha* (unroasted coffee drink)	4902201409742

JAN (Japanese article number): a 13- or 8-digit product identification number.

*Instant coffee powder was dissolved in hot distilled water (1.4% (w/v)).

† Cocoa powder was stirred into hot distilled water (4.0% (w/v)), and the supernatant was retrieved.

‡ A tea bag was immersed in 150 mL hot distilled water for 3 minutes.

The other samples were available as ready-to-drink liquid. All samples were sugar- and milk-free.

### Cell lines

HepG2 human hepatocellular carcinoma cells (Riken cell bank, Tsukuba, Japan), Caco-2 human colon cancer cells (Riken cell bank), and MCF-7 human breast cancer cells (JCRB cell bank, Osaka, Japan) were maintained in DMEM (Sigma-Aldrich) supplemented with 10% (v/v) heat-inactivated FBS and antibiotics. The DMEM bottles had been stored in the refrigerator and protected from light exposure. HepG2-XRE cells were established as follows. First, an AhR-responsive firefly luciferase reporter gene X_4_-4.27 was constructed by ligating four tandem XREs [the fragment between *Kpn I* and *Xho I* sites of the plasmid DRE_4_-GL [3]] into the pGL4.27 vector (Promega, Madison, WI), which carried the firefly luciferase gene and the hygromycin resistance gene. X_4_-4.27 was transfected into HepG2 cells using FuGENE HD (Promega), and stable transfectants were selected using hygromycin B (400 µg/mL). Using FuGENE HD, the stable transfectants were again transfected with phRL-CMV (a *Renilla* luciferase expression vector; Promega) along with pBAsi-hU6 Pur DNA vector (a plasmid carrying the puromycin-resistant gene; TaKaRa, Shiga, Japan), and maintained in the presence of both hygromycin B and puromycin (2 µg/mL). One of the resultant clones was named HepG2-XRE, and used in the reporter gene assays. By normalizing the firefly luciferase activity (which should primarily reflect AhR-dependent transcription, but also be influenced by the AhR-independent, overall transcription rate in HepG2-XRE cells) against the *Renilla* luciferase activity (which should reflect only AhR-independent transcription), it became possible to estimate AhR-dependent transcription specifically. This normalization process was also helpful in correcting for small variations of the cell number in each well, which were unavoidable even if a supposedly-equal number of cells were carefully seeded in plate wells.

### Reporter gene assays

**Figure 1:**
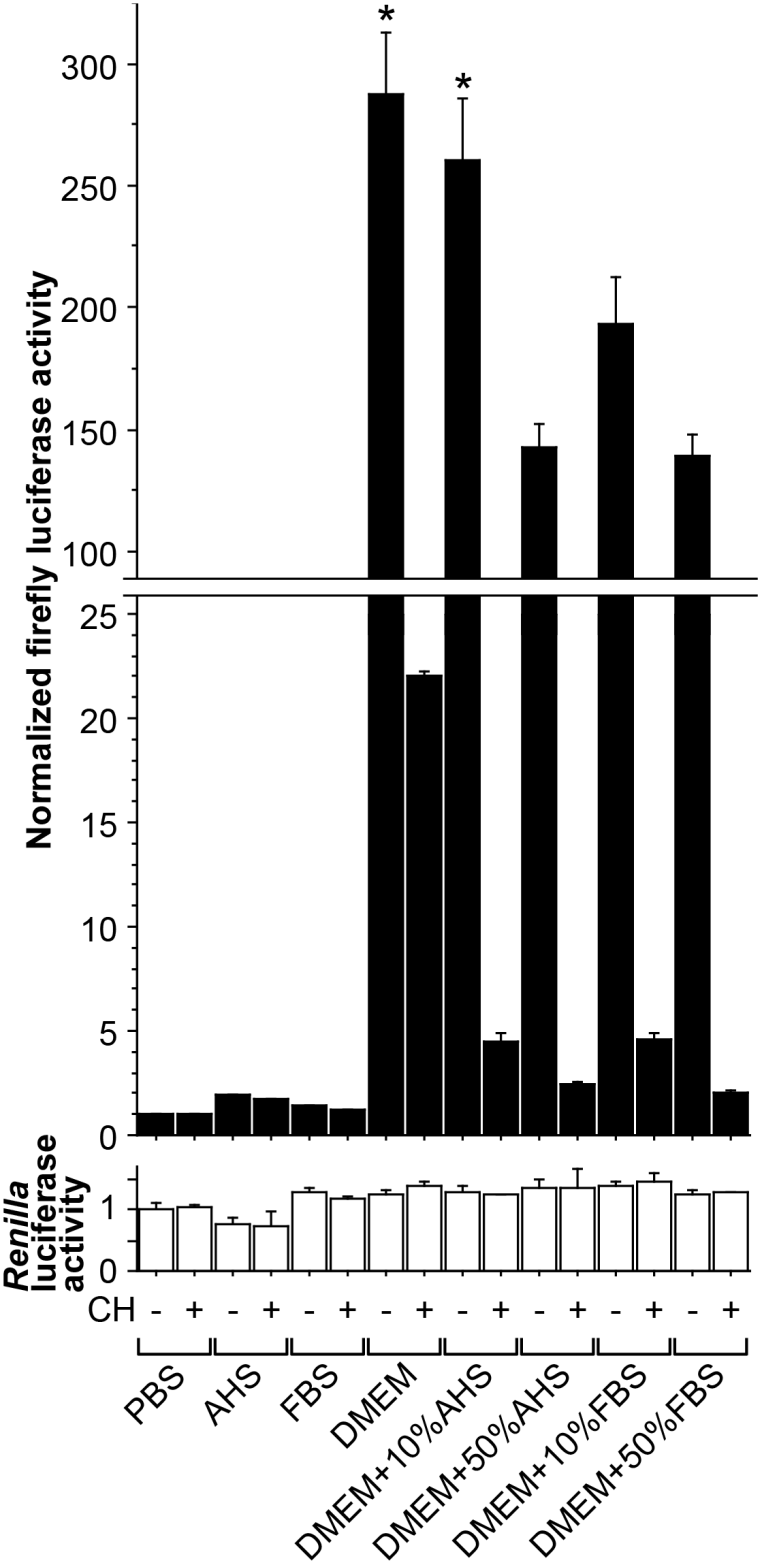
Inhibition of DMEM-induced AhR activity by serum. HepG2-XRE cells were placed for 4 h in each indicated medium, with 10 µM CH (“CH+”) or 0.1% (v/v) DMSO vehicle (“CH−”). *Renilla* luciferase activity is shown in order to deny the possibility that the observed changes in normalized firefly luciferase activity were due to extremely high or low *Renilla* luciferase activity. Error bars show standard deviations (SDs) for triplicate wells. Asterisks indicate significantly (*p*<0.05) higher normalized firefly luciferase activity than that in cells incubated in PBS without CH (1^st^ column). The mean value for the 1^st^ column was defined as 1.0.

**Figure 2:**
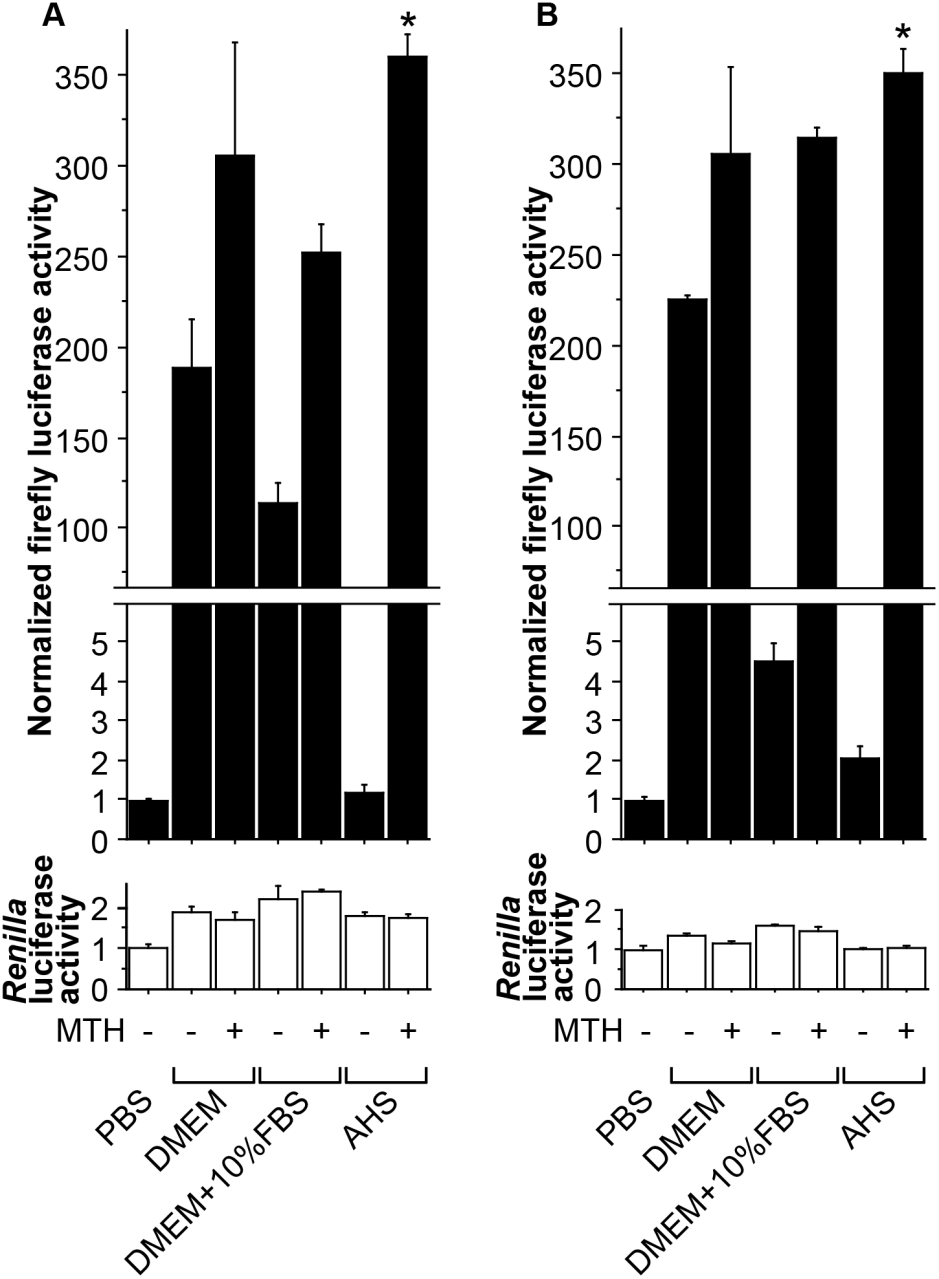
MTH-induced AhR activation in different environments. HepG2-XRE cells were incubated for 4 h in each medium, with 80 µM MTH (“MTH+”) or 0.1% (v/v) DMSO (“MTH−”). (**A**) and (**B**) were performed using the same materials and protocol, at different times. *Renilla* luciferase activity is presented for the same purpose as in Fig. 1. Error bars denote SDs for triplicate wells. Asterisks indicate significantly (*p*<0.05) higher normalized firefly luciferase activity than that in cells placed in PBS without MTH treatment (1^st^ column). The mean values for the 1^st^ column were defined as 1.0.

**Figure 3:**
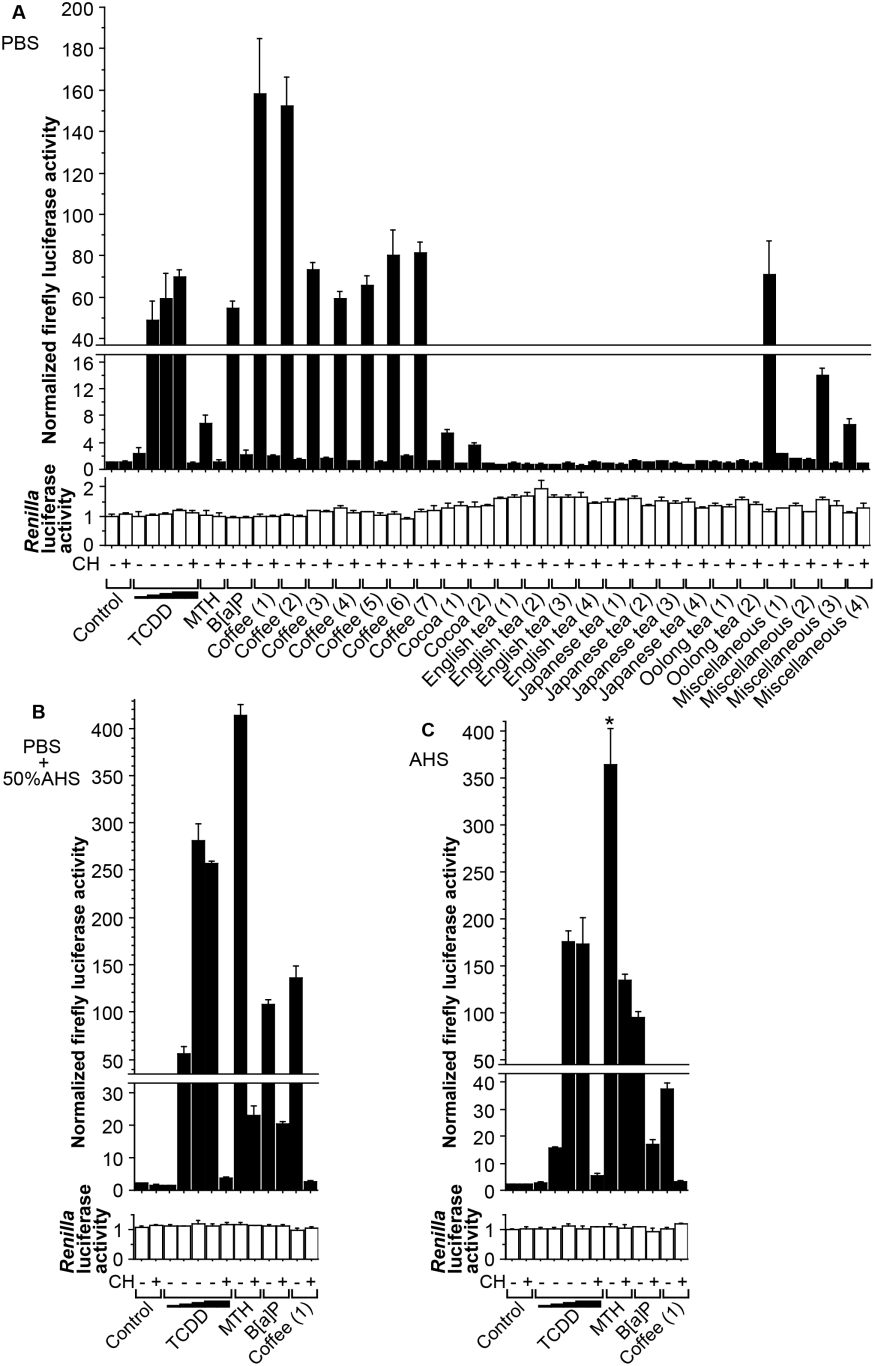
AhR activation by coffee and other beverages. HepG2-XRE cells were placed for 4 h in PBS (**A**), PBS with 50% (v/v) AHS (**B**), or AHS (**C**), with chemicals or beverages (Table 1). *Renilla* luciferase activity is shown for the same purpose as in Fig. 1. “CH+” and “CH−” indicate co-treatment with 10 µM CH and its vehicle 0.1% (v/v) DMSO, respectively. Because 0.1% (v/v) DMSO was used as a vehicle for TCDD (10^−9^, 10^−8^, 10^−7^ and 10^−6^ M), MTH (80 µM) and B[a]P (10 µM), an equal amount of DMSO was added as a control, in the samples where TCDD, MTH, or B[a]P was not added (i.e., all the columns other than the 3^rd^ through 11^th^ columns). In the samples where beverages [10% (v/v)] were not tested (i.e., the 1^st^ through 11^th^ columns), an equal amount of distilled water was added as a control. Error bars indicate SDs for triplicate wells. Experiments in (**A**), (**B**), and (**C**) were performed simultaneously, and the mean value for the control cells without CH treatment (1^st^ column) in (**A**) was defined as 1.0, in (**B**) and (**C**) as well. Asterisks indicate significantly (*p*<0.05) higher normalized firefly luciferase activity than that in the control cells without CH treatment (1^st^ column) in each graph.

One day before the experiment, one 10-cm dish of HepG2-XRE cells (∼80% confluent) were trypsinized and split equally into one 96-well Black & White tissue culture plate (Perkin-Elmer, Waltham, MA), in DMEM containing 10% (v/v) FBS and antibiotics. When more than 96 wells were needed, more cells and plates were used accordingly. On the day of the experiment, the medium was replaced with the following: in Fig. 1, PBS, AHS, FBS, or DMEM [containing no serum, 10% (v/v) AHS, 50% (v/v) AHS, 10% (v/v) FBS, or 50% (v/v) FBS], each with or without CH; in Fig. 2, PBS, DMEM [containing no serum or 10% (v/v) FBS], or AHS, each with or without MTH; and in Fig. 3, PBS [containing no serum (Fig. 3A) or 50% (v/v) AHS (Fig. 3B)] or AHS (Fig. 3C), each with or without an AhR agonist (TCDD, MTH, or B[a]P), with or without CH, and with or without a beverage sample. Four hours later [this conditioning time was chosen for three reasons: 1) longer treatment might enhance the possibility of detecting indirect AhR activation, i.e., a situation where the tested stimuli do not directly activate AhR, but instead first affect other cellular factors, which eventually leads to AhR activation; 2) consistent with the manufacturer’s description that the firefly luciferase encoded by pGL4.27 was designed to respond quickly to transcriptional induction, the optimal treatment time turned out to be 4 h in our preliminary experiments; and 3) this relatively short treatment time has been previously used in AhR reporter gene assays [10].], cells were lysed and measured for firefly and *Renilla* luciferase activities using the Dual-Luciferase Assay System (Promega) in the AB-2350 Phelios microplate luminometer (ATTO, Tokyo, Japan), and normalized firefly luciferase activity (ratio of firefly/*Renilla* luciferase luminescence) was calculated. In order to exclude the possibility that observed changes in normalized firefly luciferase activity were due to extremely high or low *Renilla* luciferase activity, *Renilla* luciferase activity was also presented in Figs. 1, 2, and 3. In each figure, normalized firefly and *Renilla* luciferase activity values were shown as fold activation relative to that of the control cells treated in PBS.

### Real-time RT-PCR

HepG2, Caco-2 and MCF-7 cells were exposed to AhR agonists or coffee in either PBS or AHS for 4 h, in 12-well plates. Three wells of cells received each treatment. Total RNA was extracted using TRIzol (Invitrogen, Carlsbad, CA, USA) and was treated with RNase-free DNase (Promega). Reverse transcription was performed using the extracted total RNA, random hexamer primers (Amersham, Uppsala, Sweden), dNTPs, RNAsin (Promega), and Moloney murine leukemia virus reverse transcriptase (Promega). The cDNA thus obtained was subjected to quantitative real-time PCR, which was performed using a LightCycler TaqMan Master kit (Roche Applied Science, Penzberg, Germany) and a LightCycler 480 (Roche Applied Science). The PCR conditions were set according to the manufacturer’s instructions. The primers for human CYP1A1 (5′-TCCAAGAGTCCACCCTTCC-3′ and 5′-AAGCATGATCAGTGTAGGGATCT-3′) and the probe used for detection (#83; Roche Applied Science) were determined using the Roche Applied Science Universal ProbeLibrary Assay Design Center (http://www.roche-applied-science.com). For each sample, real-time PCR amplification of glyceraldehyde 3-phosphate dehydrogenase (GAPDH) was performed simultaneously using the Universal ProbeLibrary Human GAPD Gene Assay (Roche Applied Science), and the CYP1A1/GAPDH ratio was calculated.

### Western blotting

HepG2, Caco-2 and MCF-7 cells were treated with AhR agonists or coffee in either PBS or AHS for 4 or 24 h, in 6-cm dishes. Cells were lysed using RIPA lysis buffer containing protease inhibitors (Santa Cruz Biotechnology, Santa Cruz, CA). The supernatants (20 µg protein) of the cell lysates were subjected to sodium dodecyl sulfate polyacrylamide gel electrophoresis (SDS-PAGE) on 7% (w/v) polyacrylamide gels, and transferred to nitrocellulose membranes. After protein transfer was confirmed with Ponceau S (Apro Science, Naruto, Japan) staining, membrane blocking was performed using Block Ace (DS Pharma Biomedical, Osaka, Japan) overnight at 4°C. The membranes were then incubated with anti-CYP1A1 antibody (sc-25304; Santa Cruz Biotechnology) and anti-β-actin antibody (sc-47778; Santa Cruz Biotechnology) in 10% (v/v) Block Ace, overnight at 4°C. After washing with Tris-buffered saline (TBS) containing 0.1% (v/v) Tween 20, the membranes were incubated with horseradish peroxidase-linked anti-mouse immunoglobulin antibody (NA931; GE Healthcare, Fairfield, CT) in 10% (v/v) Block Ace, overnight at 4°C. After washing with TBS containing Tween 20, the CYP1A1 and β-actin signals were visualized using the ECL reagent (GE Healthcare) and the LAS-4000mini imager (GE Healthcare). Signal intensities were determined densitometrically using ImageQuant TL software (GE Healthcare), and CYP1A1/β-actin signal ratios were calculated. The values in control cells were set as 1.0. The experiments were done twice independently.

### Statistical analysis

Because the variances were not equal among the control and treatment groups in our reporter gene assays and quantitative RT-PCR, statistical analyses were done using the Kruskal-Wallis test followed by Dunn’s *post hoc* test, instead of analysis of variance (ANOVA), using GraphPad Prism version 6.0 for Windows (GraphPad Software, San Diego, CA). A *p*-value<0.05 was considered as significantly different.

## Results

### Establishment of culture medium-free experimental settings

We first established a HepG2 cell line HepG2-XRE which stably expressed both an AhR-responsive firefly luciferase reporter gene X_4_-4.27, whose promoter contained four tandem XREs, and an internal control *Renilla* luciferase reporter gene phRL-CMV. HepG2-XRE cells were placed for 4 h in either PBS or DMEM containing various percentages of AHS or heat-inactivated FBS, and measured for normalized firefly luciferase activity (the ratio of firefly to *Renilla* luciferase luminescence) (Fig. 1). Compared to the normalized firefly luciferase activity in cells placed in PBS, that in cells incubated in DMEM was remarkably higher, by a factor of 288 (Fig. 1, 1^st^ and 7^th^ columns). Co-treatment with an AhR antagonist CH [11] decreased the DMEM-induced firefly luciferase expression (Fig. 1, 7^th^ and 8^th^ columns). Addition of 10% (v/v) AHS, 50% (v/v) AHS, 10% (v/v) FBS and 50% (v/v) FBS inhibited DMEM-induced AhR activation by 10%, 50%, 33% and 52%, respectively (Fig. 1, 7^th^, 9^th^, 11^th^, 13^th^ and 15^th^ columns). We suspected that this inhibition by serum might be related to our previous observation that the fold induction of AhR activity by addition of MTH was invariably higher in cells treated in AHS than in cells treated in FBS-containing DMEM [3]. Thus, we tested how MTH induced AhR activity in HepG2-XRE cells incubated in DMEM with or without 10% (v/v) FBS or in AHS, repeating the same experiments at different times (Fig. 2, A and B). In Fig. 2, just as in Fig. 1, cells placed in DMEM exhibited clearly higher firefly luciferase activity than those in PBS, by a factor of 189 (Fig. 2A, 1^st^ and 2^nd^ columns), and 226 (Fig. 2B, 1^st^ and 2^nd^ columns). However, to what extent serum inhibited the DMEM-induced AhR activity varied considerably from experiment to experiment: while addition of 10% (v/v) FBS reduced DMEM-induced AhR activity by 33% in Fig. 1 (7^th^ and 13^th^ columns) and 40% in Fig. 2A (2^nd^ and 4^th^ columns), it decreased DMEM-induced AhR activity by 98% in Fig. 2B (2^nd^ and 4^th^ columns). Depending on whether the resultant “basal” AhR activity in cells placed in DMEM containing 10% (v/v) FBS was high or low, MTH elicited AhR activity only “modestly” (Fig. 2A, 4^th^ and 5^th^ columns) or “markedly” (Fig. 2B, 4^th^ and 5^th^ columns). This inhibitory effect of FBS was uncontrollable and unpredictable even if the experiments were done using the same materials and protocol. Even when FBS suppressed DMEM’s AhR agonist activity substantially (Fig. 2B, 4^th^ and 6^th^ columns), the “basal” AhR-stimulating activity of DMEM with 10% (v/v) FBS was still higher than that of AHS. Thus, for the reproducibility and sensitivity of the experiment, we chose to use either PBS or serum instead of the commonly used DMEM plus FBS, because the intrinsic AhR-stimulating activity of PBS or serum was negligible, judging from the effect of CH (Fig. 1).

### Induction of AhR-responsive reporter gene activity by coffee

When HepG2-XRE cells were incubated for 4 h in PBS with the known AhR agonists and various beverages (Table 1), all the coffee samples markedly induced firefly luciferase activity, which was clearly inhibited by addition of CH (Fig. 3A). Some coffee beverages exhibited higher AhR agonist activity than 10^−6^ M TCDD. Two cocoa samples stimulated AhR weakly, and three out of the four miscellaneous beverages elicited AhR activation. “Coffee (1)” which induced AhR activation most in PBS among the beverage samples (Fig. 3A) was tested for its AhR-stimulating activity in PBS with 50% (v/v) AHS (Fig. 3B) and in AHS (Fig. 3C), as well. While this coffee sample stimulated AhR more strongly than TCDD (10^−6^ M), MTH (80 µM) and B[a]P (10 µM) in PBS (Fig. 3A), it activated AhR to a lesser extent than TCDD and MTH in PBS with 50% (v/v) AHS (Fig. 3B). In AHS, TCDD, MTH and B[a]P stimulated AhR more than coffee (Fig. 3C). Taken together, the efficacy of various AhR activators could be different, depending on the environment where cells were treated with each activator. However, it could be safely concluded that coffee was definitely an AhR stimulator.

### Induction of CYP1A1 expression by coffee

**Figure 4:**
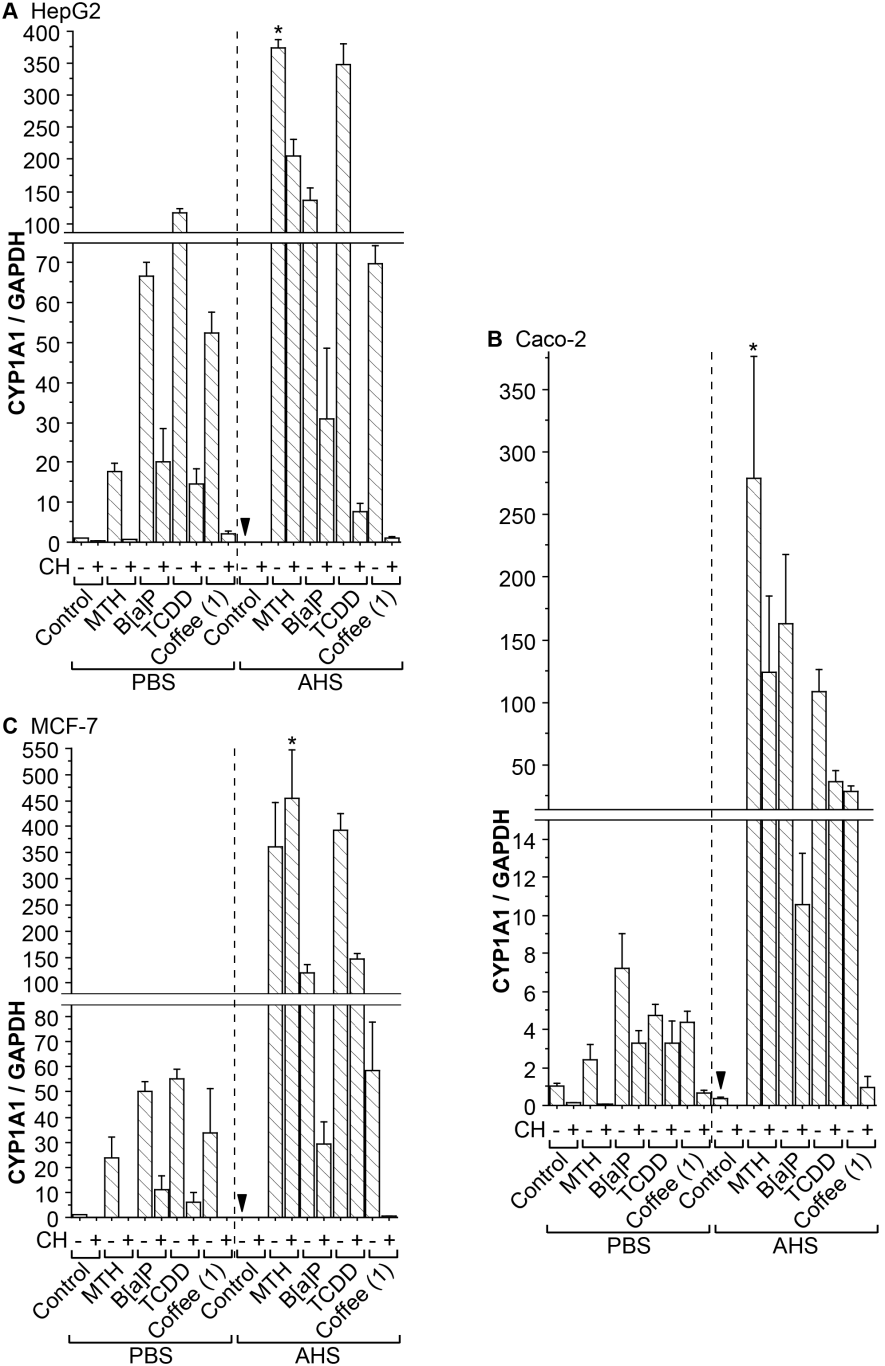
Induction of CYP1A1 mRNA by coffee. HepG2 (**A**), Caco-2 (**B**), and MCF-7 (**C**) cells were incubated for 4 h in either PBS or AHS with each sample added as in Fig. 3, except that TCDD was added only at 10^−6^ M. Error bars indicate SDs for triplicate samples. The mean values for the control cells treated with DMSO in PBS were defined as 1.0. Asterisks indicate statistically significant differences (*p*<0.05) from the control cells incubated in AHS (arrowheads).

We next checked the expression of CYP1A1, a representative AhR-responsive gene in HepG2, Caco-2 and MCF-7 cells. First, CYP1A1 mRNA levels were measured after 4-h treatment with different AhR agonists and “Coffee (1)” (Fig. 4). There was a tendency that coffee enhanced CYP1A1 expression, albeit to a lesser extent than MTH, B[a]P, or TCDD in both PBS and AHS, in the three cell lines. In some cases, CH did not effectively inhibit CYP1A1 induction by MTH. However, the coffee-induced CYP1A1 expression was always suppressed profoundly by co-treatment with CH.

**Figure 5:**
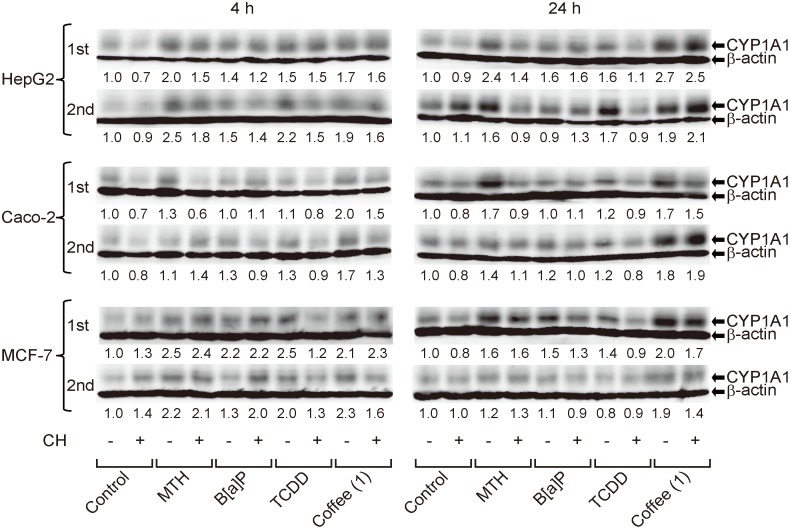
Induction of CYP1A1 protein by coffee. HepG2, Caco-2, and MCF-7 cells were cultured for 4 or 24 h in AHS with each sample added as in Fig. 3, except that TCDD was added only at 10^−6^ M. Because CYP1A1 protein was undetectable in cells incubated in PBS for either 4 or 24 h, only the results from cells placed in AHS are shown. In order to clearly visualize the CYP1A1 signals (56 kDa), the immunoblots in which the simultaneously detected β-actin bands (43 kDa) were overexposed are presented. Two experiments (“1^st^” and “2^nd^”) were performed independently. The relative band intensities (CYP1A1/β-actin), which were determined by defining the value observed in control cells as 1.0, are shown below each immunoblot.

Using Western blotting, we also assessed CYP1A1 protein levels in HepG2, Caco-2 and MCF-7 cells, after 4- or 24-h treatment, in either PBS or AHS. When these cells were placed in PBS with or without AhR agonists or coffee for either 4 or 24 h, the expression of CYP1A1 protein was undetectable (data not shown). Therefore, only the results from cells treated in AHS are shown in Fig. 5, where the experiments were performed twice independently (“1^st^” and “2^nd^”). The magnitudes of CYP1A1 protein induction by AhR ligands were not so remarkable as those of CYP1A1 mRNA induction (Fig. 4), and were somewhat variable between the 1^st^ and 2^nd^ Western blot experiments (Fig. 5). Also, the AhR antagonistic effects of CH were not consistently observed. As for the known AhR agonists, MTH (80 µM) tended to enhance CYP1A1 protein expression in all three cell lines and TCDD (10^−6^ M) seemed to consistently induce CYP1A1 protein only in HepG2 cells, but B[a]P (10 µM) was not a consistent inducer in any of these cell lines. Curiously, coffee stimulated CYP1A1 protein expression in all three cell lines, more reliably than the known AhR ligands tested here.

These experiments demonstrated that coffee upregulated CYP1A1 protein as well as mRNA levels in cells placed in AHS. In cells incubated in PBS, coffee elevated CYP1A1 mRNA, but it was impossible to tell whether coffee induced CYP1A1 protein because its expression was undetectable by Western blotting.

## Discussion

In this study, after establishing HepG2-XRE, a HepG2 cell line stably expressing an AhR-responsive reporter gene, we first showed that it would be safer to test AhR activation with cells placed in PBS or pure serum rather than in FBS-containing DMEM, whose intrinsic AhR agonistic activity could interfere with the AhR activation assays. We then demonstrated that coffee not only induced AhR-dependent firefly luciferase activity in HepG2-XRE cells, but also enhanced expression of CYP1A1 mRNA and protein in HepG2, Caco-2, and MCF-7 cells. Of note, in Figs. 1, 2, 3, and 4, even an obvious difference in normalized firefly luciferase activity or CYP1A1 mRNA expression was in many cases not regarded as statistically significant, presumably for two reasons: 1) because we dealt with a relatively large number of groups in each experiment, it might be difficult to detect statistically significant differences; and 2) due to the unequal variances among groups, we adopted the Kruskal-Wallis test, whose statistical power was lower than that of ANOVA. Thus, this should be kept in mind when looking at these figures.

AhR-responsive reporter gene assays and analysis of CYP1A1 expression have been commonly used for detection or estimation of AhR-activating xenobiotics in environmental samples [12], [13]. The basic principle of our reporter gene assay was the same with that used in previous studies, and our quantitative RT-PCR and Western blot analyses of CYP1A1 expression were based on the standard techniques. However, the following three new attempts were made in our experiments.

First, and most importantly, we created our own AhR-responsive reporter cell line (HepG2-XRE) to avoid a pitfall that could theoretically happen to the experiments using reporter cells carrying only an AhR-responsive reporter gene: in such experiments, an increase in reporter gene expression does not necessarily indicate specific upregulation of AhR-mediated transcription, because there is a possibility that the tested sample may be enhancing the overall gene transcription rate. We therefore developed HepG2-XRE cells by stably transfecting not only a firefly luciferase gene bearing four XREs in its promoter (X_4_-4.27), but also a *Renilla* luciferase gene with no XRE (phRL-CMV), into HepG2 cells. In the assay using HepG2-XRE, the *Renilla* luciferase activity was used for normalization of the firefly luciferase activity, as a reference to correct for the effect of a sample on the overall transcription rate as well as the cell number in each culture plate well. Importantly, when HepG2-XRE was created, X_4_-4.27 and phRL-CMV were transfected sequentially, but not simultaneously. In general, when plasmids are transfected into mammalian cells, at least a portion of introduced plasmids are cut, and then ligated into a linearized concatemer, because double-strand breaks in different plasmid molecules are glued together through the non-homologous end-joining DNA repair pathway [14]. Therefore, if X_4_-4.27 and phRL-CMV had been co-transfected, they would have formed a heteroligated concatemer and then cointegrated into the same site of the genome [15], resulting in a situation in which the expression of *Renilla* luciferase from phRL-CMV could have been influenced by the XREs present on X_4_-4.27. Therefore, we transfected these two genes independently using different selectable markers, i.e., hygromycin and puromycin resistance genes. In retrospect, correction of the firefly luciferase activity against the *Renilla* luciferase activity had little, if any, impact on the results of our reporter gene assays, because the tested conditions did not substantially change *Renilla* luciferase activity (Figs. 1, 2, and 3). Nonetheless, in order to precisely evaluate how samples induce the AhR-dependent, but not overall, gene transcription, it would be prudent not to skip this standardization process.

Secondly, we routinely used a specific AhR antagonist CH, for the purpose of confirming that the elevation of firefly luciferase activities or CYP1A1 expression levels was indeed ascribed to AhR stimulation. However, it turned out that while CH did exert its antagonist activity in some experiments (e.g. Fig. 3A), it did not always reduce AhR activation effectively in others (e.g. Fig. 5). In fact, a previous study reported that CH strongly antagonized AhR activation induced by halogenated aromatic hydrocarbons (e.g. TCDD), but did not affect AhR stimulation by other AhR ligands (e.g. PAHs) [16]. Our results were somewhat inconsistent with this study: for example, in Fig. 3A, CH inhibited AhR activation elicited even by non-halogenated ligands such as MTH and B[a]P, whereas in Fig. 4B, CH inhibited TCDD-induced CYP1A1 expression only modestly. Thus, although our results agree that CH does not always act as a complete inhibitor of AhR activity, it seems that much remains to be tested regarding the conditions in which CH fully exerts its AhR antagonistic effect.

Thirdly, we tried unconventional experimental settings in order to obtain sensitive results. It has been reported that light-exposed culture medium exhibits AhR-stimulating activity because 6-formylindolo[3,2-*b*]carbazole (FICZ), a photoproduct formed by UV irradiation of tryptophan, acts as an AhR ligand [9]. Although our DMEM had been protected from light during storage in the laboratory, it clearly showed AhR-stimulating activity (Figs. 1 and 2), suggesting the possibility that it might have been exposed to light during transport and/or that FICZ or other AhR agonists could have been synthesized in DMEM through unknown mechanisms. Consistent with a previous report that serum reduced potencies of AhR ligands [17], the AhR-stimulating activity of our DMEM was inhibited by addition of serum (FBS or AHS) (Fig. 1). This observation, along with our previous finding that induction of cellular AhR activity by MTH could be detected more sensitively in AHS than in FBS-supplemented DMEM [3], prompted us to try AHS as a cell culture environment in this study. Nonetheless, theoretically, AHS might not be the best possible environment, given the possibility that the endogenous AhR ligands that should be contained in AHS might interfere with the experiments, even though their physiological concentrations were insufficient for AhR activation as was speculated in the case of bilirubin [1]. Therefore, besides AHS, we also tried PBS, which contained only common electrolytes and thus should be devoid of any AhR-stimulating activity, as a cell culture environment. After all, although it turned out that the basal intrinsic AhR-stimulating activity of AHS was only slightly higher than that of PBS (Figs. 1 and 2), AhR-dependent firefly luciferase or CYP1A1 mRNA expression was induced by AhR agonists to different extents in PBS and in AHS (Figs.3 and 4). In particular, MTH elicited AhR activation much more strongly in AHS than in PBS, which might be accounted for at least partly by the solubility of MTH. Thus, the results are quite dependent on which culture environment is chosen when experiments are performed.

Regarding the results of Western blotting, in PBS, the expression of CYP1A1 was undetectable even after addition of AhR agonists or coffee (data not shown), which was at least in line with the report showing that induction of CYP1A1 protein by TCDD was reduced in PLHC-1 cells after serum withdrawal [18]. By contrast, in AHS, the basal expression was clearly observed, but the induction by AhR agonists was obviously less pronounced than that of CYP1A1 mRNA and sometimes was not observed at all (Fig. 5), which might seem consistent with a previous report that serum reduced the potency of TCDD with regard to its action of inducing CYP1A1 protein [17]. However, the same report also revealed that this potency reduction was due to the inhibitory action of serum on cellular uptake of TCDD, which was incongruous with our RT-PCR data where CYP1A1 mRNA was nicely induced by TCDD presumably after sufficient uptake of TCDD into cells. In light of the different extents of CYP1A1 mRNA and protein induction by AhR ligands (Figs. 4 and 5), CYP1A1 translation and/or posttranslational degradation rates may be important in regulating CYP1A1 protein levels. Although an example has been shown in which CYP1A1 expression is modulated at the posttranslational level, by heavy metals (Hg^2+^, Pb^2+^, and Cu^2+^) [19], much remains to be elucidated regarding the translational and posttranslational regulation of CYP1A1. There is a possibility that CYP1A1 protein might be degraded rapidly when cells are placed in PBS, but further studies are necessary to test it.

The discrepancy between the firefly luciferase activity and the CYP1A1 protein expression in regard to the magnitude of induction by AhR agonists or coffee (Figs. 3C and 5) should be discussed as well. Although the exact reasons for this discrepancy are unclear, the structural characteristics of firefly luciferase and CYP1A1 genes and proteins could be involved. Even though CYP1A1 is well known as an AhR-responsive endogenous gene, its responsiveness may be inferior to that of the artificial firefly luciferase encoded by X_4_-4.27, whose parental plasmid pGL4.27 was designed exclusively for the purpose of sensitive detection of transcriptional upregulation through the transcription factor-binding sites inserted into its promoter. Also, we need to take into consideration the fact that in the first place we selected the clone that expressed both firefly and *Renilla* luciferase proteins very strongly as HepG2-XRE.

Collectively, in cell-based AhR activation assays, it is difficult to determine a single “one-fits-all” cell culture environment. Whatever cell types are used, it may be necessary to try different analysis methods to evaluate AhR activation (AhR-responsive reporter gene assays, CYP1A1 RT-PCR and Western blotting, etc.), using different cell culture milieus (PBS, AHS, etc.).

Regarding the effects of coffee on AhR-mediated gene expression, as was mentioned above, the sensitivity for detecting coffee-induced gene expression was not similar among the experimental methods: while firefly luciferase activity and CYP1A1 mRNA expression were clearly induced by coffee (Figs. 3 and 4), the level of CYP1A1 protein was only mildly increased by coffee in cells placed in AHS (Fig. 5). For some reason, coffee, which was the weakest inducer of CYP1A1 mRNA (Fig. 4), was probably the most consistent CYP1A1 protein inducer in AHS (Fig. 5). Possibly, unlike the impurity-free AhR agonists purchased from chemical manufacturers, coffee might contain some substances [e.g. heavy metals [19]] which would slow down the degradation of CYP1A1 protein, but further experiments are needed to test this possibility.

Although roasted coffee beans do contain PAHs [5], it is impossible at this stage to tell exactly which compounds in coffee beverages are responsible for AhR activation and to what extent such compounds are absorbed from the gut, delivered to the liver via portal circulation, and eventually distributed to various organs. Regardless of whether the AhR agonists contained in ingested coffee stay in the gastrointestinal tract or enter the systemic circulation, it should be kept in mind that there is a limitation in our study which precludes generalization: although coffee stimulated AhR in HepG2, Caco-2, and MCF-7 cells, they are immortal carcinoma cell lines maintained *ex* *vivo* in the laboratory conditions. Therefore, our findings should not be immediately extrapolated to the cells under physiological *in* *vivo* conditions. Certainly coffee beverages, upon being ingested, get in direct contact with the epithelial cells of the upper gastrointestinal tract, but it remains to be verified whether coffee indeed activates AhR even in these cells.

As described above, coffee-induced AhR activation has not yet been confirmed in human cells *in* *vivo*, but our observation may still raise a concern that coffee drinking might have detrimental effects on human health, because AhR is also known as the dioxin receptor, which transmits signals of xenobiotic toxins. Epidemiologically, there have been conflicting reports regarding whether coffee consumption is beneficial or harmful. For example, in 2012, a very large-scale study reported that coffee drinking was associated with lower mortality [20], whereas in 2013 another report attracted attention from around the world because it claimed that heavy coffee drinking was linked to a higher mortality risk in people under the age of 55 [21]. In these studies, it is highly likely that the effects of the AhR-stimulating activity in coffee on human health might be obscured due to various bioactive substances (e.g. caffeine) contained in coffee. Regarding specifically the AhR-stimulating activity of coffee, this property may actually have favorable influences on health, because some dietary AhR ligands might be beneficial for the prevention and treatment of inflammatory bowel disease, metabolic syndrome, etc [22]. On the other hand, the AhR agonist activity of coffee may also be exerting harmful effects, which could be exemplified by the controversial link between coffee consumption and bladder cancer [23], the risk of which has been shown to be increased in those occupationally exposed to PAHs [24]. When coffee-induced AhR activation is actually verified in human cells *in* *vivo*, effects of coffee drinking on health will be assessed more properly and strictly in the future.

In conclusion, by performing reporter gene assays and CYP1A1 RT-PCR and Western blot analyses in either PBS or AHS instead of commonly used culture medium, we have demonstrated that coffee activates AhR in cultured cells. This observation may help elucidate the effects of coffee drinking on human health.
